# A system dynamics model of clinical decision thresholds for the detection of developmental-behavioral disorders

**DOI:** 10.1186/s13012-016-0517-0

**Published:** 2016-11-25

**Authors:** R. Christopher Sheldrick, Dominic J. Breuer, Razan Hassan, Kee Chan, Deborah E. Polk, James Benneyan

**Affiliations:** 1 Department of Pediatrics, Tufts Medical Center, 800 Washington Street #854, Boston, MA 02111 USA; 2 Healthcare Systems Engineering Institute, Northeastern University, 360 Huntington Ave, Boston, MA 02115 USA; 3 Department of Health Policy and Administration, University of Illinois, Chicago, School of Public Health, 1603 West Taylor Street, Chicago, IL USA; 4 Dental Public Health and Information Management, University of Pittsburg, 381 Salk Hall, Pittsburgh, PA 15261 USA

**Keywords:** Clinical decision-making, Threshold, Screening, System dynamics, Behavioral disorders, Developmental disorders

## Abstract

**Background:**

Clinical decision-making has been conceptualized as a sequence of two separate processes: *assessment* of patients’ functioning and application of a *decision threshold* to determine whether the evidence is sufficient to justify a given decision. A range of factors, including use of evidence-based screening instruments, has the potential to influence either or both processes. However, implementation studies seldom specify or assess the mechanism by which screening is hypothesized to influence clinical decision-making, thus limiting their ability to address unexpected findings regarding clinicians’ behavior. Building on prior theory and empirical evidence, we created a system dynamics (SD) model of how physicians’ clinical decisions are influenced by their assessments of patients and by factors that may influence decision thresholds, such as knowledge of past patient outcomes. Using developmental-behavioral disorders as a case example, we then explore how referral decisions may be influenced by changes in context. Specifically, we compare predictions from the SD model to published implementation trials of evidence-based screening to understand physicians’ management of positive screening results and changes in referral rates. We also conduct virtual experiments regarding the influence of a variety of interventions that may influence physicians’ thresholds, including improved access to co-located mental health care and improved feedback systems regarding patient outcomes.

**Results:**

Results of the SD model were consistent with recent implementation trials. For example, the SD model suggests that if screening improves physicians’ accuracy of assessment without also influencing decision thresholds, then a significant proportion of children with positive screens will not be referred and the effect of screening implementation on referral rates will be modest—results that are consistent with a large proportion of published screening trials. Consistent with prior theory, virtual experiments suggest that physicians’ decision thresholds can be influenced and detection of disabilities improved by increasing access to referral sources and enhancing feedback regarding false negative cases.

**Conclusions:**

The SD model of clinical decision-making offers a theoretically based framework to improve understanding of physicians’ behavior and the results of screening implementation trials. The SD model is also useful for initial testing of hypothesized strategies to increase detection of under-identified medical conditions.

**Electronic supplementary material:**

The online version of this article (doi:10.1186/s13012-016-0517-0) contains supplementary material, which is available to authorized users.

## Background

Evidence suggests that the profusion of clinical practice guidelines has had variable effects on the behavior of physicians [1–3] and that the field of medicine is marked by high levels of practice variation, which is often cited as a sign of waste [4, 5]. From their perspective, physicians report that standardized guidelines are often too rigid to apply to the complex presentations of individual patients [6, 7], and the Institute of Medicine/National Academy of Medicine concurs that clinical guidelines are not a “one-size-fits-all approach” [3, 6, 7]. Thus, challenges to the implementation of evidence-based practices often arise from the behavior of the participants involved. As others have concluded, efforts to improve healthcare would benefit from greater understanding of how physicians make decisions and what would motivate them to change [8, 9].

Screening for developmental-behavioral conditions, which affect up to 20% of children, offers a prime example. Despite evidence for the importance of early intervention, less than one third of children with such disabilities are typically identified in primary care [10, 11]. US organizations such as the American Academy of Pediatrics (AAP) have recommended evidence-based screening instruments to improve detection [12, 13], and use of screeners has increased sharply in recent years [14]. In addition, numerous studies have been conducted to understand the effect of implementing screening in primary care [15]. Results suggest that case disposition is seldom determined purely by screening scores—physicians and other decision-makers typically play a large role. Table 1 lists 16 implementation trials for developmental or behavioral screening in primary care. Among the ten trials that reported relevant data, referral rates among children with positive screens ranged from 10 to 86%, suggesting that a substantial proportion of children with positive screens are not referred. In addition, implementation of screening often resulted in unexpected changes in referral rates. In the nine trials with relevant data, changes in referral rates ranged widely—from a significant decline to increases of several times the original referral rate. The potential for a decline is consistent with a recent meta-analysis of depression screening trials that found an average 3% decline in referrals [16]. Thus, screening can lead to unexpected outcomes.

**Table 1:** Implementation trials of developmental and behavioral screening

Reference	Sample size	Setting	Type of screening	Physicians’ recognition of disorders among children with positive screens (%)	Referral rate among children with positive screens (%)	Change in recognition rate attributable to screening (Relative risk)	Change in referral rate attributable to screening (Relative risk)
Earls et al. 2009 [70]	526	Pediatric primary care	Developmental screening		60.2% (95% CI 49.8–70.0%)		
Schonwald et al. 2009 [71]	759	Pediatric primary care	Developmental screening			1.27 (95% CI 0.91–1.76)	1.18 (95% CI 0.72–1.93)
King 2010 [72]	Not reported	Pediatric primary care	Developmental screening		62%^a^		
Guevara et al. 2013 [73]	2103	Pediatric primary care	Developmental screening		86.4% (95% CI 80.2–91.3%)		1.94 (95% CI 1.47–2.58)
Dawson and Camp 2014	418	Pediatric community health centers	Developmental screening		74.4% (95% CI 66.0–81.7%)		
Thomas et al. 2016 [74]	54	Family medicine clinic	Autism, depression and developmental screening		10.3% (95% CI 2.2–27.4%)		0.65 (95% CI 0.23–1.84)
Murphy et al. 1996 [75]	379	School-based clinics	Behavioral health screening		62.5% (95% CI 45.8–77.2%)		4.64^a^
Gall et al. 2000 [76]	383	school based clinic	Behavioral health screening		80.8% (95% CI 67.5–90.4%)		
Hacker et al. 2006 [77]	1668	Pediatric primary care	Behavioral health screening				1.98^a^
Stevens et al. 2008 [78]	878	Pediatric primary care	Behavioral health screening	64.9% (95% CI 58.8–70.7%)		1.09 (95% CI 0.86–1.37)	
Wintersteen 2010 [53]	3040	Pediatric primary care	Suicide Risk			4.33 (95% CI 2.5–7.6)	4.33 (95% CI 2.5–7.6)
Kuhlthau et al. 2011 [79]	Not reported	Pediatric primary care	Behavioral health screening			3.04	
Berger-Jenkins et al. 2012 [80]	229	Pediatric primary care	Behavioral health screening			0.89 (95% CI 0.66–1.12)	0.63 (95% CI 0.41–0.95)
Rausch et al. 2012 [81]	636	Pediatric primary care	Adolescent Depression		58.0% (95% CI 43.2–71.8%)		
Jonovich and Alpert-Gillis 2014 [54]	356	Pediatric primary care	Behavioral health screening	25.2% (95% CI 18.3–33.1%)	43.4% (95% CI 35.1–51.9%)	1.19 (95% CI 0.74–1.83)	2.38 (95% CI 2.15–5.75)
Romano-Clarke et al. 2014 [82]	600	Pediatric primary care	Behavioral health screening		49.5% (95% CI 39.9–59.2%)	0.89 (95% CI 0.57–1.41)	0.85 (95% CI 0.43–1.69)

^a^Indicates insufficient data to calculate confidence interval or to include in meta-analysis

Without an appropriate theory of clinicians’ behavior, results such as these are counterintuitive at best. As many have argued, theory is essential to effective implementation [17–19]. In this case, a coherent theory is needed to understand why primary care clinicians behave as they do and what would motivate them to change [9].

Decision analysis can be extremely useful for understanding screening and referral decisions [20–23]. For example, the “general assessment and decision-making model” posits that two independent elements underlie any decision—one’s *assessment* of the situation and the *threshold* used to determine whether the amount and weight of evidence is sufficient to justify a given decision [23]. In decision analysis, a *decision threshold* represents a point of indifference where the costs and benefits of referral are perceived to be in balance. If the appraisal of a patient’s risk or symptom severity meets or exceeds the physician’s *decision threshold*, action will be taken; if not, no action will be taken. Thus, referrals are made when the benefits of action are perceived to outweigh the costs. In contrast, when the likely costs of referral are perceived to outweigh the benefits, no referral will be made.

An important insight of threshold models is that variation in referral decisions across providers, studies, or contexts can be conceptualized as resulting from differences in the accuracy of assessment, differences in decision thresholds, or both. Moreover, threshold models suggest that assessment and decision thresholds are each influenced by different factors functioning at the level of the decision-maker or in the decision-maker’s context. For example, physicians’ accuracy in assessing patients’ symptoms may be influenced by factors that include the provider’s skills and training, time available for examination, and access to relevant information. In contrast, decision thresholds are likely to be influenced by a separate set of factors, “such as emotions, regret of omission versus commission, financial incentives” [8], and other factors that influence perceptions regarding the costs and benefits of referral [24–26].

The explanatory power of threshold models can be expanded through integration with signal detection models, where *decision thresholds* are often referred to as “cut scores.” Whereas decision analytic models help to describe how a physician’s threshold may change in response to perceived changes in the costs and benefits of referrals [20, 27], signal detection models demonstrate the impact of thresholds on the frequency of true positive (TP), false positive (FP), true negative (TN), and false negative (FN) results. By integrating these two approaches, we can learn more about the physicians’ perspectives on the clinical significance of threshold changes not only for individuals, but also for the populations of patients they serve.

To better understand physicians’ decision thresholds and their implications for patient populations, we developed a system dynamics (SD) model that integrates insights from both decision analysis and signal detection theory. SD models are sets of coupled differential and other relational equations designed to simulate the interdependent behavior of processes over time [28]. Just as a model airplane offers a way to understand the structure of an aircraft, SD modeling’s “causal loop” and “stock-and-flow” diagrams provide intuitive visualizations of complex systems. Similar to using a wind tunnel to efficiently test hypotheses regarding a prototype airplane’s performance, simulations based on SD models are designed to help develop insight and hypotheses about longitudinal behaviors, especially for systems that involve feedback loops and time delays. SD modeling has been recommended by the National Institutes of Health for studying policy resistance [29, 30] and systems engineering more broadly has been advocated for improving healthcare processes [31, 32], including by the Institute of Medicine and National Academy of Engineering in several joint publications [33].

Below, we describe the development and structure of our SD model, its application to implementation trials of developmental-behavioral screening, and a series of virtual experiments designed to explore novel interventions to improve detection of developmental-behavioral disorders in primary care settings.

## Methods

### Development and structure of the SD model

**Fig. 1:**
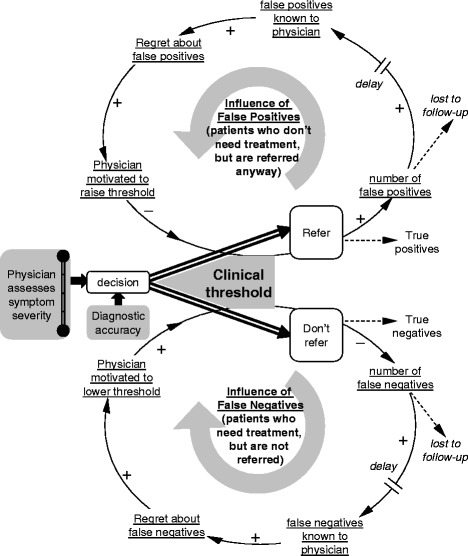
Overview of dynamic thresholds theory

Our SD model focuses on how knowledge of past patient outcomes influences decision thresholds (see Fig. 1). In this model, a physician assesses the symptom severity of sequential patients with a given level of accuracy. When the physician’s perception of symptom severity exceeds his or her decision threshold, the patient is referred. Otherwise, the patient is not referred. Referral decisions can be correct (TP or TN) or incorrect (FP or FN). After a referral decision, patients are sometimes *lost to follow-up*, but other times their status becomes *known to the referring physician* after a delay. Over time, information regarding the results of some past referral decisions ultimately feeds back and influences the physician’s decision threshold for future patients. Known errors can lead to *regret.* Increased regret about FPs may motivate a physician to raise his or her decision threshold (i.e., requiring greater symptomatology to trigger a referral), thereby reducing the number of patients referred. Conversely, increased regret about FNs may motivate a lowering of the decision threshold (i.e., requiring lesser symptomatology to trigger a referral), thereby increasing the number of patients referred. Changes in decision thresholds therefore are influenced by the type of error (FP or FN) for which a physician has more regret at that moment.

Any factors that influence physicians’ perceptions of a clinical outcome’s probability or impact may also influence his or her decision threshold. The model accounts for such factors through three primary sets of variables: one that reflects the accuracy of the initial assessment, a second that reflects physicians’ perceptions regarding the relative costs of incorrect referrals (FP) and failures to refer (FN), and a third that reflects the amount and timeliness of feedback regarding FP and FN errors. Different practice-level interventions might plausibly have different effects on each set of variables. For example, introduction of high-quality screening instruments is likely to increase accuracy by providing physicians with additional information about patients’ symptoms. However, suggested “cut scores” may not change physicians’ decision thresholds if they do not influence their perceptions of the costs and benefits of referral. In contrast, addition of new referral sources—such as co-located mental health clinicians—may favorably influence physicians’ perceptions of the costs and benefits of referral. Finally, systematic feedback systems may improve physicians’ knowledge of past errors [34, 35]. As has been noted, “For many decisions we make, we are more likely to receive feedback about some outcomes than about others, and thus we must operate under conditions of asymmetric partial feedback” [36]. The practice of medicine is no exception. Patients often do not follow through with referral appointments [37, 38], physicians seldom receive feedback regarding patient outcomes [39], and feedback regarding FP and FN cases often is delayed [40]. On the other hand, evidence suggests that learning of adverse events resulting from specific medications influences physicians’ decision thresholds for prescribing those medications but not others [41]. SD models are uniquely designed to model feedback [28], and our SD model explicitly includes feedback about FP and FN errors occurring with different probabilities and delays.

### Model structure

**Fig. 2:**
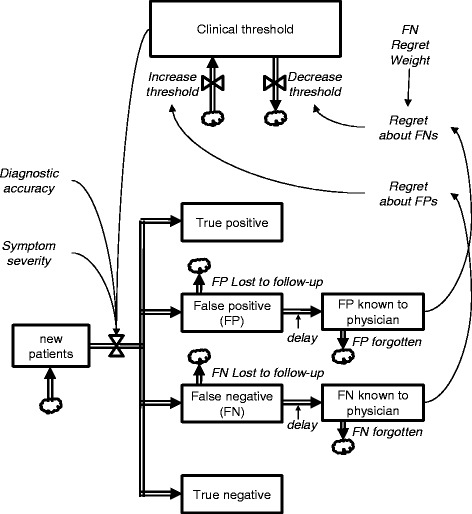
Summary stock-and-flow structure of system dynamics (SD) model

Figure 2 depicts the model's structure; a full working version is included as an Additional File 1, and instructions are included as Additional File 2. The model begins when the physician assesses patients’ symptoms and makes referral decisions, thus yielding TP, FP, TN, and FN results. Physicians’ assessments and patients’ diagnostic status are modeled by drawing random numbers from a bivariate normal distribution, which we graph using three dimensions:

1. “True” *symptom severity* is assumed to be normally distributed in the population, consistent with evidence suggesting the results of behavioral instruments can be modeled as normal distributions [42]. In our model, prevalence of developmental-behavioral disorders is assumed to be 15% [43]. Therefore, if a child’s “true” *symptom severity* falls in the extreme 15% of the population, the patient is classified as having a disorder.
2. *Physician’s perceptions of symptom severity* is also assumed to be normally distributed, and its correlation (rho) with *“true” symptom severity* represents the *accuracy* of the physician’s assessment. High correlations indicate that physicians’ assessments display little error, while low correlations indicate the opposite.
3. A third axis represents the frequency of children at each point on the plane described by the first two axes.

When a random observation is drawn, the value for “true” symptom severity determines whether or not the child has a disorder, while the value for the physician’s perception of symptom severity is compared to a *decision threshold* to determine whether or not the child is referred. Together, these classifications determine which of the four branches each patient follows: TP, FP, TN, or FN Calculations for the bivariate normal distribution are included in Additional File 3.

Our model assumes that loss to follow-up for each type of outcome can vary over a wide range with different time lags, and physicians experience regret when either FN or FP errors become known. The relative regret associated with each type of error is represented by the *FN regret weight,* which can either raise or lower the impact of a known FN case compared to a known FP case. Finally, we assume that when physicians experience regret regarding FP or FN errors, they adjust their decision thresholds to reduce the likelihood of repeating that type of error, thereby affecting future referral decisions.

Note that our model includes several simplifying assumptions. For example, physicians’ assessments are assumed to be unidimensional and continuous, consistent with previous models [44] and similar to the Children’s Global Assessment of Functioning by which clinicians rate children’s overall functioning on a 100-point scale [45]. Additionally, we assume that the influence of known outcomes diminishes as a linear function of time as memory fades and past experiences become less relevant. We do not mean to imply that physicians actually perceive patients’ symptoms and outcomes in precisely this manner, only that this can be a parsimonious way to model their influence on referral processes. Consistent with *regret theory* [46, 47] and the approach of a prior SD model of policy threshold oscillations [48], our model focuses only on the influence of errors and does not explicitly incorporate correct decisions. As originally described by Peirce [49], the net benefit associated with a decision can be defined as a function of the number and marginal utility of TP and FP outcomes. This formulation serves as the foundation for decision-curve analysis [50], and it has been adapted by Tsalatsanis et al. [46] to focus instead on FP and FN errors. In their formulation, the marginal utility of a FP is equivalent to the disutility from unnecessary treatment, while the marginal utility of a FN is equivalent to the disutility from failure to treat. Focusing on errors in this way has the advantage of being more parsimonious than accounting for the full expression of net benefits, and it is consistent with previous descriptions of the influence of regret on decision thresholds [51].

### Internal validation

To ensure that all equations were specified correctly and as intended, the entire model was replicated by a team of healthcare systems engineering graduate students from Northeastern University. Performance of the original and replicated models was identical. We also conducted a series of tests to ensure that the model performs as expected. For example, we tested the model under an assumption of perfect accuracy to ensure that the expectation of perfect sensitivity and specificity are met, and we calculated the FN regret weight that should yield equivalent sensitivity and specificity based on regret theory [46] and tested whether the model behaved accordingly.

### Model calibration

We included several conceptually relevant variables despite the fact that empirical data were sparse or unavailable, including those variables that determine the probability and timeliness of feedback for each outcome. To develop values for these variables, we used a calibration process by which values of unobserved variables are estimated by adjusting them until output fits observed data [52]. Because relevant time series data on pediatricians’ behavior were unavailable, we calibrated our model to results from a recent systematic review on the sensitivity and specificity with which physicians detect developmental and behavioral disorders [11]. This systematic review found that when pediatric providers identify behavioral disorders in general practice, specificity typically far exceeds sensitivity, indicating a reluctance to commit FP errors and an inclination to favor positive predictive value [11]. A bivariate meta-analysis of reported results yielded physician sensitivity = 38.7% (95% CI 26.1–53.1%) and specificity = 87.7% (95% CI 80.2–92.5%). Estimates for *FN regret weight* and loss to follow-up and delay for both FP and FN results were adjusted until the difference between model output and the above sensitivity and specificity values were minimized, using sum of squared differences as a criterion. Because the model is underspecified, meaning that multiple sets of parameters can meet calibration criteria, we created a series of plausible scenarios that include ranges for each parameter. Scenarios included estimates of *FP loss to follow-up* that ranged from 10 to 60%, delay for FN results that ranged from 1 to 5 times the delay for FP results, and regret for a FN case that ranged from 1 to 5 times the regret for a FP case. For each scenario, FN *loss to follow-up* was adjusted until the model’s average sensitivity and specificity most closely approximated the results of the systematic review. Additional scenarios were developed by calibrating to the upper and lower bounds of the 95% confidence intervals for sensitivity and specificity based on the systematic review.

While all scenarios were included in sensitivity analyses, a base case model was chosen to represent the most plausible scenario. For this case, we assumed a FN regret weight = 3; i.e., that pediatricians regret a FN (missing a true case) three times as much as a FP (referring a patient who does not benefit). We base this assumption on the observation that a large percentage of pediatric referrals for mental health treatment do not result in services. Pediatricians’ continued willingness to refer patients under such conditions suggests acceptance of a number of FPs for every child who ultimately receives services. Furthermore, we assumed that physicians’ knowledge of FP results, although imperfect, is greater than their knowledge of FN results. Because there are seldom formal systems to detect missed mental health diagnoses and to report such errors back to the pediatrician, knowledge of FNs is likely to be rare. In contrast, pediatricians are more likely to learn that a referred patient was found to be ineligible for services, either directly from the treatment provider or from the patient at a subsequent visit. Thus, we specified loss to follow-up for FP = 20% and loss to follow-up for FN = 73%, and we assumed that delay for learning about FP results (50 patient visits) was much less than delay for learning about FN results (250 patient visits).

### Application to developmental-behavioral screening

To test the model’s utility for exploring real-world results, we compared model predictions to the results of published studies described in Table 1, which were not included in model development. To estimate summary values for the referral rate among children with positive screens and the change in referral rates attributable to screening implementation, meta-analyses of published results were conducted using the *metan* command in Stata version 12.

We modeled the implementation of screening by altering physicians’ accuracy (rho; i.e., the correlation between true symptom severity and the physician’s perception of symptom severity) from 0.65 (which yields a Receiver Operating Characteristics [ROC] curve that includes sensitivity and specificity of approximately 75%) to 0.85 (which yields an ROC curve that includes sensitivity and specificity of approximately 85%). Note that this analysis accounts only for increased accuracy of assessment attributable to screening instruments; no influence on thresholds is assumed. Thus, we hypothesized that our model would under-estimate the results of most screening trials, which often include additional interventions such as training or co-located services. Change in identification rates was estimated by calculating the proportion of children referred in the base case model and then recalculating the proportion of children referred after adjusting rho. The proportion of children with positive screening results was represented by calculating the ratio of children referred to the number who would score positive with rho = .85 if a threshold were chosen based on Youden’s index (i.e., the threshold that maximizes the sum of sensitivity and specificity, as is typical of developmental-behavioral screeners). All scenarios identified during model calibration were tested. Model results therefore reflect a plausible range of parameters across trials.

### Virtual experiments

To explore novel interventions designed to improve detection of developmental-behavioral disorders, we altered the following parameters, one at a time, to investigate the impact on performance:

- Experiment #1: increased accuracy. To simulate the introduction of a high-quality screening questionnaire, we increased assessment accuracy from 0.65 to 0.85, as described above.
- Experiment #2: increased FN regret weight. Various factors may alter physicians’ regret regarding FN versus FP errors (i.e., the regret ratio). For example, referrals for mental health services can be time-consuming for both providers and patients, and concern about stigma may provide additional barriers. Convenient and non-stigmatizing follow-up services, possibly through collaborative, co-located mental health care, may reduce patients’ burden, making FP results more tolerable. Reducing physicians’ regret for FPs relative to their regret for FNs may induce lower decision thresholds.
- Experiment #3: decreased loss to follow-up for FNs. Formal systems to provide systematic feedback have been recommended to improve patients’ mental health outcomes [34]. We simulated such a solution by reducing loss to follow-up for FNs, which may motivate physicians to lower decision thresholds.
- Experiment #4: increased accuracy, increased FN regret weight, and decreased loss to follow-up (combined intervention). In experiment #4, we tested the combined effect of all three parameter changes.

For each virtual experiment, average sensitivity and specificity were calculated based on results from 3000 patients estimated after an initial run of 2000 patients to minimize the influence of initial threshold values. For experiments #2–4, the magnitude of change was set to yield sensitivity equivalent to experiment #1.

## Results

### Model calibration

Our base case yielded an average sensitivity of 39.6% and an average specificity of 92.4%. For subsequent analyses, variation was explored across the full range of scenarios described above.

### Application to developmental-behavioral screening

**Fig. 3:**
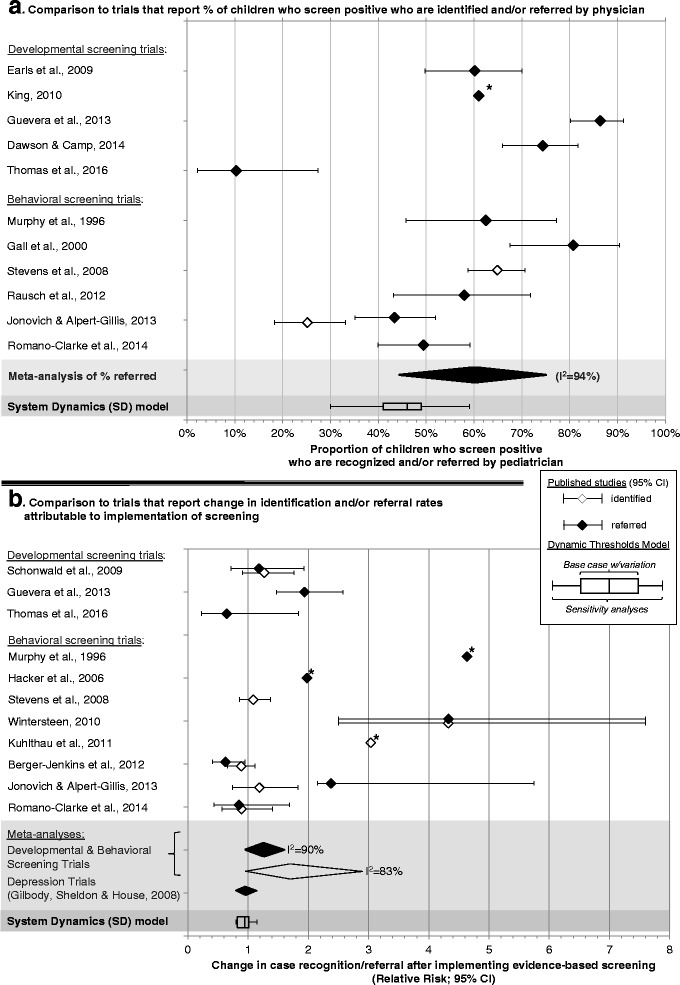
Comparison of model output to results of screening implementation trials regarding: **a** identification and referral rates among children who screen positive, and **b** change in identification and referral rates attributable to screening

Figure 3a displays results from published studies and from our model regarding referral rates among children with positive screens. Among the nine studies with sufficient data, meta-analysis indicated that an average pooled proportion of 60% (95% CI 44–75%) of children with positive screens were referred. However, results ranged widely from 10 to 86%, and meta-analysis revealed statistically significant heterogeneity among studies (*p* < 0.0005; *I* ^2^ = 94%), suggesting that there is likely to be considerable clinical and/or methodological differences among trials. In comparison, the base case SD model yielded a proportion of 42%, with values from sensitivity analyses ranging from 30 to 59%. Thus, SD model predictions fell toward the lower end of the range of results of published trials (many of which included interventions beyond screening), as expected.

Figure 3b depicts the change in referral rates attributable to screening. Among the seven trials reporting relevant data, the Mantel-Haenszel pooled relative risk was 1.67 (95% CI 0.96–2.9). However, results ranged widely, from a 37% decline in referrals attributable to the implementation of screening to a 333% increase, and meta-analysis revealed statistically significant heterogeneity among studies (*p* < 0.0005; *I* ^2^ = 90%). We also include results from a recent meta-analysis of depression screening trials, which found an average 3% decline in referral rates (RR = 0.97; 95% CI = 0.81–1.18) [16]. In comparison, the base case SD model yielded a 6% decrease in referrals attributable to the implementation of screening, with values from sensitivity analyses ranging from a 20% decline to a 15% increase. Thus, while the range of values predicted by our SD model included the results of the meta-analysis of depression screening trials and overlapped with the low end of the range of results reported in trials of developmental-behavioral screening instruments, the significant heterogeneity again suggests that there are likely to be considerable clinical and/or methodological differences among trials (see Additional File 4 for more detailed description of included studies).

To further explore this heterogeneity, we recalibrated the model to be more consistent with two studies with extreme results. For example, one study found that referrals increased 4.3 times over baseline when screening for suicide risk was implemented [53]. To explore plausible parameter values that might explain this result, we first calibrated the model to reflect the 0.8% referral rate reported at baseline by changing the FN regret ratio to one. As above, we then modeled implementation of screening by changing rho from 0.65 to 0.85, which resulted in 2.7 times the number of referrals found at baseline. To explore what factors might explain the even larger change in referral rates reported in the study, we also changed the FN regret ratio from 1 to 5.5, reflecting the observation that pediatricians may be more likely to regret FN results for suicide than for other developmental-behavioral disorders. This change resulted in 4.6 times the referrals found at baseline, thereby approximating study results.

A second example consisted of a study of pediatric mental health screening which found referrals to increase 2.4 times over baseline [54]. To explore plausible parameter values that might explain this result, we first calibrated the model to reflect the 10.3% referral rate reported at baseline in the study by changing the rate at which FN were lost to follow-up from 73 to 80%. As above, we modeled implementation of screening by changing rho from 0.65 to 0.85, which resulted in a slight reduction in the number of referrals compared to baseline (RR = 0.99). To explore factors that might explain the even larger change in referral rates reported in the study, we focused on the paper’s report that significant mental health services were provided in the primary care setting in addition to screening. We hypothesized that provision of co-located mental health services would decrease the perceived costs associated with a FP result, thus increasing the FN regret ratio (because the denominator (FP) decreases as the numerator (FN) remains constant). However, even an extreme FN regret ratio of 100 was insufficient to achieve this result. A complementary hypothesis is that if co-located mental health clinicians offer preventive interventions, then in effect the prevalence of children who may benefit may be higher than for standard interventions. Changing model prevalence from 15% to 25% while holding other variables constant indeed increased referral rates, after which an FN regret ratio of 8 was sufficient to replicate study results.

### Virtual experiments (#1–4)

**Fig. 4:**
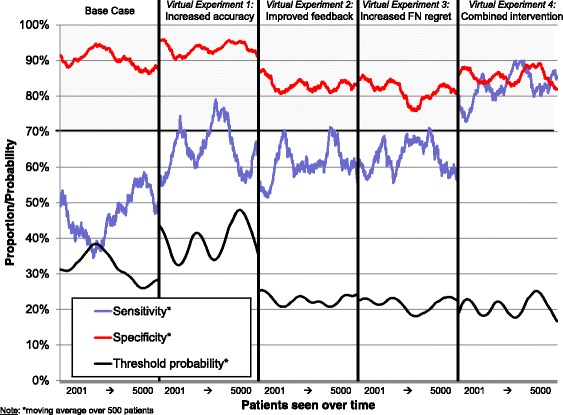
Results of virtual experiments #1–4

Table 2 summarizes parameter values for the four virtual experiments; Fig. 4 summarizes results. Sensitivity and specificity that fall above the dark horizontal line at 70% exceed consensus regarding minimum standards for developmental-behavioral screening instruments [13]. Decision thresholds are represented as threshold probabilities, which refer to the probability a child who scores at the threshold has a disorder.

**Table 2:** Parameter values for virtual experiments

Virtual experiment	Model parameters			
	False positives (FP) lost to follow-up	False negatives (FN) lost to follow-up	Assessment accuracy (rho)	Regret ratio (FN/FP)
Base case	20%	73%	0.65	3
1. Increased accuracy	20%	73%	*0.85*	3
2. Improved feedback	20%	*20%*	0.65	3
3. Increased FN regret	20%	73%	0.65	*10*
4. Combined intervention	20%	*20%*	*0.85*	*10*

Values in italics indicate change compared to baseline

Designed to simulate pediatricians’ use of screening instruments, virtual experiment 1 regarding assessment accuracy yielded average sensitivity = 60% and specificity = 94%. Although 85% sensitivity and specificity were possible if physicians selected a threshold according to Youden’s index, our model predicted that physicians would respond to the implementation of screening by raising their thresholds, thus achieving a different point on the same ROC curve (see “increased assessment accuracy” in Fig. 4). By lowering decision thresholds, our two other simulated interventions achieved similar improvements in sensitivity, but at the cost of decreased specificity. In experiment #2, increasing the regret ratio from 3 to 10 achieved average sensitivity = 57% and specificity = 83%. In experiment #3, reducing loss to follow-up for FNs from 73 to 20% achieved average sensitivity = 61% and specificity = 81% (see “increased FN regret” in Fig. 4).

Changing all three parameters simultaneously (see “combined intervention” in Fig. 4) simulated a combined intervention in which assessment accuracy was increased, regret about false negatives was increased relative to regret about FPs, and feedback about FNs was improved. In this scenario, average sensitivity and specificity were estimated at 84 and 83%, respectively.

In a set of sensitivity analyses, we conducted the same four experiments using a baseline model that assumed 60% (rather than 20%) of FP results were lost to follow-up. To calibrate the model to achieve similar sensitivity and specificity, the FN regret ratio was reduced to 2 (rather than 3). Using this baseline model, the results of all four virtual experiments yielded very similar patterns to those described above (see Additional file 5 for results). Together, these virtual experiments suggest that strategies that go beyond encouraging pediatricians to use formal screening tools may be helpful for improving detection rates.

Although our virtual experiments focused on average values, at least one additional observation is notable: threshold probability, sensitivity, and specificity oscillated over time in all conditions. These oscillations appeared to be larger in some conditions (i.e., virtual experiment #1) than in others (i.e., virtual experiment #2).

## Discussion

Many frameworks, such as the socioecological model and the Consolidated Framework for Implementation Research (CFIR), highlight the importance of considering intrapersonal variables alongside context. The SD model of thresholds for clinical decision-making proposes specific mechanisms to describe the relationship between the individual and his or her setting. For a wide range of parameter values, the assumption that screening directly increases accuracy while having an ambiguous effect on thresholds yields predictions that are broadly consistent with implementation trials of developmental-behavioral screening, including that not all positive screens will be referred and that changes in referral rates attributable to the implementation of screening may range widely depending on context and even decline in some circumstances. The key insight is that depending on the perspective of the decision-maker, increased accuracy may reduce FP errors and FN errors to different degrees. If only the former, the number of referrals (which include both TP and FP results) will decrease. Although our ability to fully validate the model is limited by a lack of study-specific data on relevant parameters (such as physician regret and rates of loss to follow-up associated with FP and FN errors), the studies that diverged most markedly from our model provide interesting case studies. For example, one trial that resulted in a very large increase in both identification and referrals (falling well beyond the range predicted by our model) also was the only study in our sample to focus on suicide [53]—an outcome for which physicians are likely to regret FNs far more than for other behavioral conditions. A separate trial that reported a very large change in referrals also included provision of significant mental health services in addition to screening [54], which our model predicts will influence decision thresholds. Thus, the model offers plausible hypotheses to explain heterogeneity.

Our SD model also offers structured hypotheses regarding novel multi-level strategies to improve detection of developmental-behavioral disorders. Similar to frameworks that highlight the role of screening in the context of clinical decision-making [55], our model suggests ways in which interventions might influence not only physicians’ accuracy but also the thresholds they use to make clinical decisions. Thus, multi-level interventions that provide feedback regarding patients’ outcomes and address physicians’ perceptions of the costs and benefits of referrals may yield better outcomes than evidence-based screening implemented as an isolated strategy. For example, a range of recent policy initiatives may influence the key variables in our model. Computerized adaptive testing shows promise for increasing the accuracy of screening instruments [56]. Other initiatives may influence physicians’ decision thresholds, such campaigns to reduce stigma [57], improved connections between primary care and mental health resources [58], and preventive services that have lower costs but that offer benefits to a greater proportion of children than intensive treatments [59].

It is important to highlight several limitations. As in all models, simplifying assumptions were necessary to produce a workable, understandable model. For example, our model does not consider downstream effects of physicians’ decisions on other service providers. Physicians’ regret is likely to be informed by a range of factors that are beyond the scope of our model, including personal (e.g., personality, training), interpersonal (e.g., influence from other physicians), and contextual (e.g., quality of mental health resources, chance of audit or lawsuit), each of which could be the focus of a more detailed model.

Similarly, we did not explicitly model cognitive processes that influence decision-making. For example, research on hindsight bias demonstrates the influence of memory on behavior [60]. Research on dual process models, which posit both explicit and implicit processes for decision-making, documents a range of systematic biases in decision-making, such as anchoring effects and loss aversion as highlighted by cumulative prospect theory [61]. They also suggest that humans often rely on the “gist” of information to make decisions rather than rational deliberations regarding probabilities and utilities [62]. While our model represents thresholds as probabilities, physicians may be more likely to consider whether the amount, weight, or “gist” of evidence is sufficient to justify a decision. Previous work suggests that threshold models can incorporate elements of dual process theories [51], and this presents a promising avenue for advancing our SD model.

Despite its simplifying assumptions, our SD model helps to extend theory on clinical decision-making in important ways. Whereas most models of decision-making focus on single decisions made in isolation, our SD model recognizes that physicians make similar decisions repeatedly over time. Recent research suggest that humans may approach iterated decisions very differently than single decisions in isolation [63], for example, by underweighting small probabilities when making decisions based on experience rather than overweighting small probabilities as is typically found in experimental paradigms that rely on explicit descriptions of probabilities and outcomes [64, 65]. By allowing for the possibility that individuals may learn from past experience, iterated decision models suggest that the extent and type of feedback physicians receive may have strong implications for their clinical decision-making.

### Implications for implementation science

Our SD model offers new insights into screening and clinical decision-making that have wider implications for implementation science. For example, our model suggests that the behavior of physicians can influence the expected effect of evidence-based screening instruments and clinical practice guidelines, even if they are behaving rationally and in the best interests of their patients. As many have argued, it may be more useful to attribute errors to the design and management of patient care processes than to the limitations of individual providers [66, 67], and therefore systems-level interventions may be needed to improve detection. Thus, simply convincing physicians to adopt screening instruments may fail to account for the complexity of clinical decision-making and may even lead to unintended consequences, such as attenuating the potential impact of screening instruments and even diminishing physicians’ trust in expert guidelines. Quoting the Framework for Analyzing Adoption of Complex Health Innovations, it is reasonable to ask, “What are the harmful effects of an external ‘push’ (such as a policy directive or incentive) for a particular innovation when the system is not ready?” [68]. Situations when seemingly obvious solutions do not work as well as intended or lead to unintended consequences are common in implementation and have long been described in the SD literature as “policy resistance” [69]. SD models have the potential to highlight participants’ role in implementation efforts and the potential downstream consequences if their reactions are ignored.

Our SD model also has implications for assessment in implementation research. If success depends in large part on local decision-makers, then greater attention to their behavior is warranted. This includes actual decisions made, how they are influenced by perspectives on costs and benefits and knowledge of past outcomes, and how these variables may change over time. For example, closer attention to physicians’ decision thresholds, the individual and contextual factors that influence them, and whether and how they change over time may be helpful for understanding screening trials. Such data will be critical to further develop our SD model and calibrate it to describe clinical care.

Finally, our model highlights how the use of simulation modeling to integrate and apply theory to practical questions in implementation science can lead to novel insights. For example, our model suggests that decision thresholds may oscillate over time, even given stable parameters. Such behavior is common in complex systems characterized by feedback with differential delays [28] and oscillations have long been observed in decision thresholds in public policy [48]. Whereas the literature on practice variation typically focuses on inter-individual or inter-group difference in medical care, oscillations in decision thresholds may represent a source of intra-individual variation. Thus, it is possible that physicians with identical characteristics working in identical contexts may nevertheless exhibit different behavior if they are at different points in the same process of threshold oscillation. While model results cannot provide proof, they do offer structured, causal hypotheses that can be systematically explored and refined in future research.

## Conclusions

The sheer complexity of implementation research presents significant scientific challenges [69]. Explicitly designed to improve thinking about systems, SD simulation models offer tools to assist in understanding the implications of this complexity. As an example, our SD model offers plausible hypotheses to explain otherwise counterintuitive results of screening implementation trials, including the consistent observation that physicians refer only a fraction of children who screen positive and that changes in referral rates attributable to the implementation of screening vary markedly across trials, but are often modest. In addition, the model suggests that interventions designed to influence physicians’ decision thresholds may be important for improving detection rates of developmental-behavioral disorders. While preliminary, results suggest that by modeling explicit causal theories, SD simulations can complement prominent frameworks in implementation science, such as CFIR.

## Additional files

Additional file 1: System dynamics model of clinical decision thresholds - Vensim file. (MDL 75 kb)
Additional file 2: Brief instructions for running system dynamics model in Vensim. (DOCX 850 kb)
Additional file 3: Calculations relevant to bivariate normal distribution. (XLS 237 kb)
Additional file 4: Table S1. Supplementary information regarding developmental and behavioral screening trials. (DOC 36 kb)
Additional file 5: Figure S1. Sensitivity analyses for virtual experiments. (PPTX 385 kb)
