# Supplementary material for: A system dynamics model of clinical decision thresholds for the detection of developmental-behavioral disorders
Source: Implement Sci. 2016 Nov 25;11:156. doi: 10.1186/s13012-016-0517-0 (PMC5123221; doi:10.1186/s13012-016-0517-0)
Supplement: Additional file 2: — Brief instructions for running system dynamics model in Vensim. (DOCX 850 kb) [file 13012_2016_517_MOESM2_ESM.docx]

**To begin a simulation of the SD model:**

- Download and install “Vensim PLE” software from Vensim website^[[1]](#footnote-1)^ (<http://www.vensim.com/freedownload.html>). The software is free, but you will need to register.
- Open Vensim.
- Open the model.
- Click “SyntheSim” button to run model (see figure below); adjust sliders to alter parameters.

“SyntheSim” button


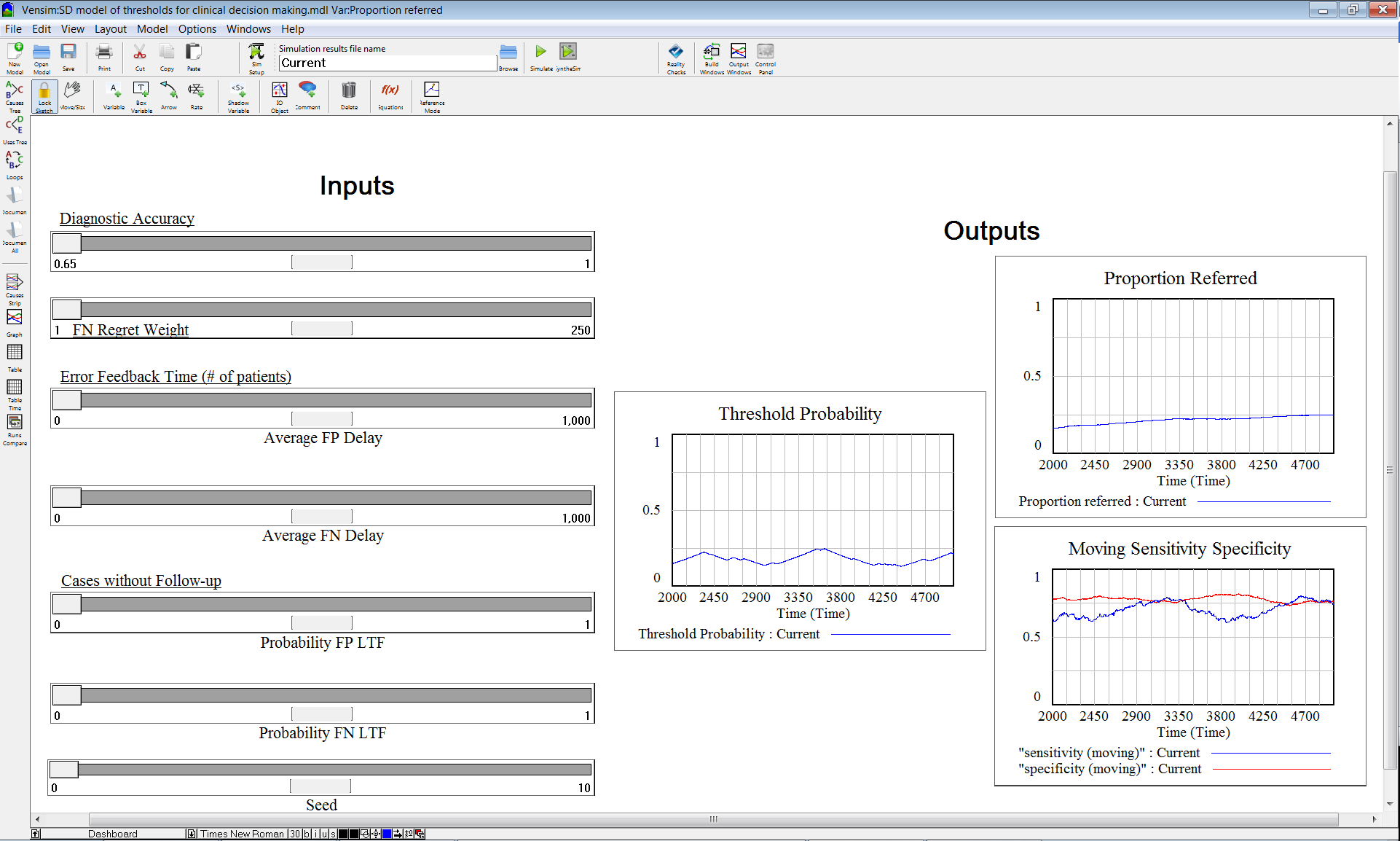


**Running the simulation**

- Inputs can be changed using the sliders on the left. Current values for each input appear in the center of the slider.
- Inputs include:
  - Diagnostic accuracy: correlation between “true symptoms” and “physician’s assessment of symptoms” (initial value = 0.65)
  - FN Regret Weight: physician’s perspective on the impact of a False Negative (FN) result as compared to a False Positive (FP) result (initial value = 3)
  - Average FP delay: average time before a False Positive (FP) result becomes known to the physician (initial value = 50)
  - Average FN delay: average time before a False Negative (FN) result becomes known to the physician (initial value = 250)
  - Probability FP LTF: probability that a False Positive (FP) result will be lost to follow-up (LTF) (initial value = 0.60)
  - Probability FN LTF: probability that a False Negative (FN) result will be lost to follow-up (LTF) (initial value = 0.73)
  - Seed: Determines the random order in which patients present (i.e., different “seeds” specify different random orders) (initial value = 0)
- Outputs include:
  - Threshold probability: probability of disorder for a child assessed at the decision threshold
  - Proportion referred: proportion of children who score above the threshold and are therefore referred
  - Sensitivity: proportion of children *with* disorders who are correctly classified by assessment
  - Specificity: proportion of children *without* disorders who are correctly classified by assessment


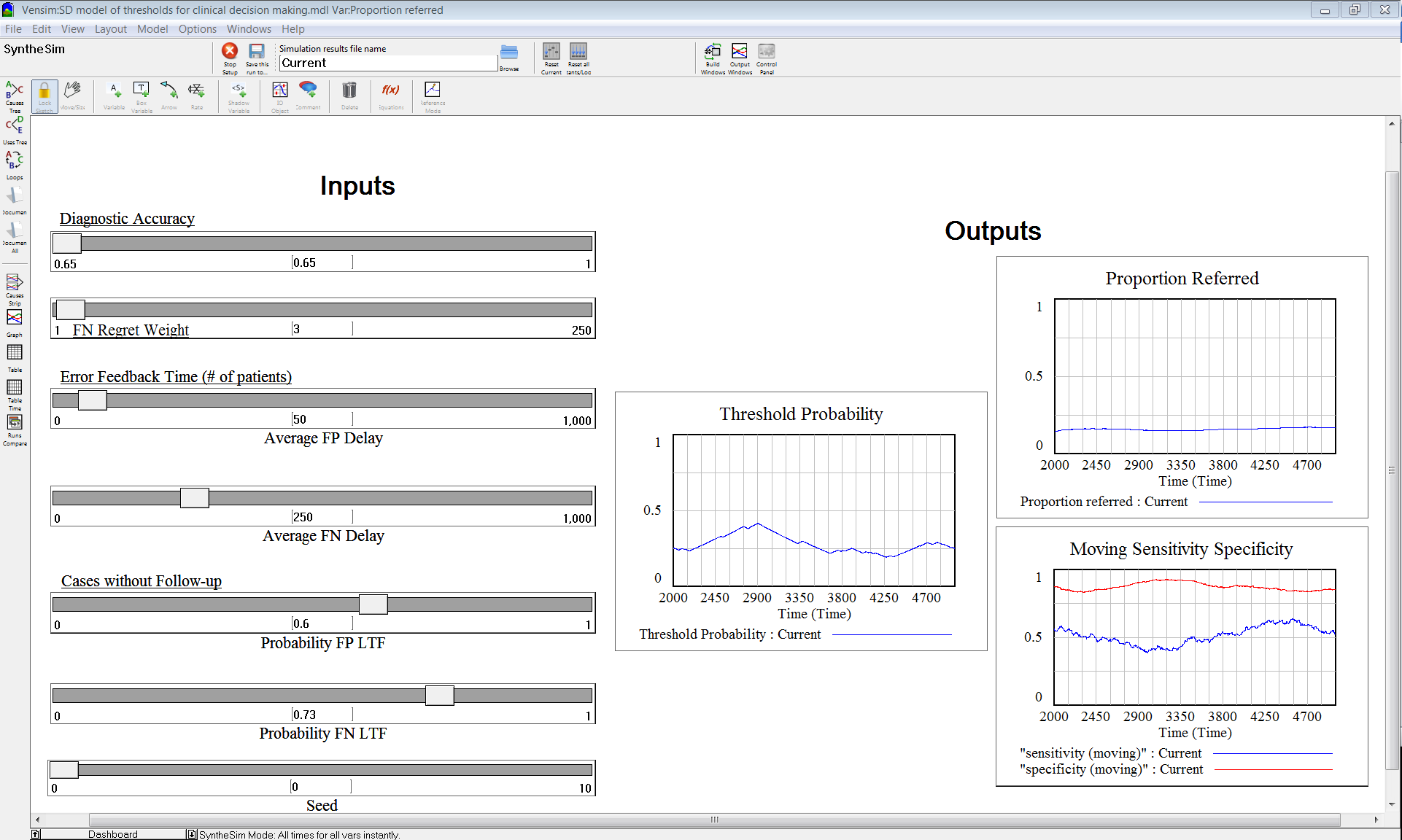


Current values

Sliders

**Examining model structure and equations**

- To examine structure of full model, change from “Dashboard” to “Model” view using control at lower left (see figure below).
- To view an equation for a part of the model, click “equations” button and then any variable name on the screen


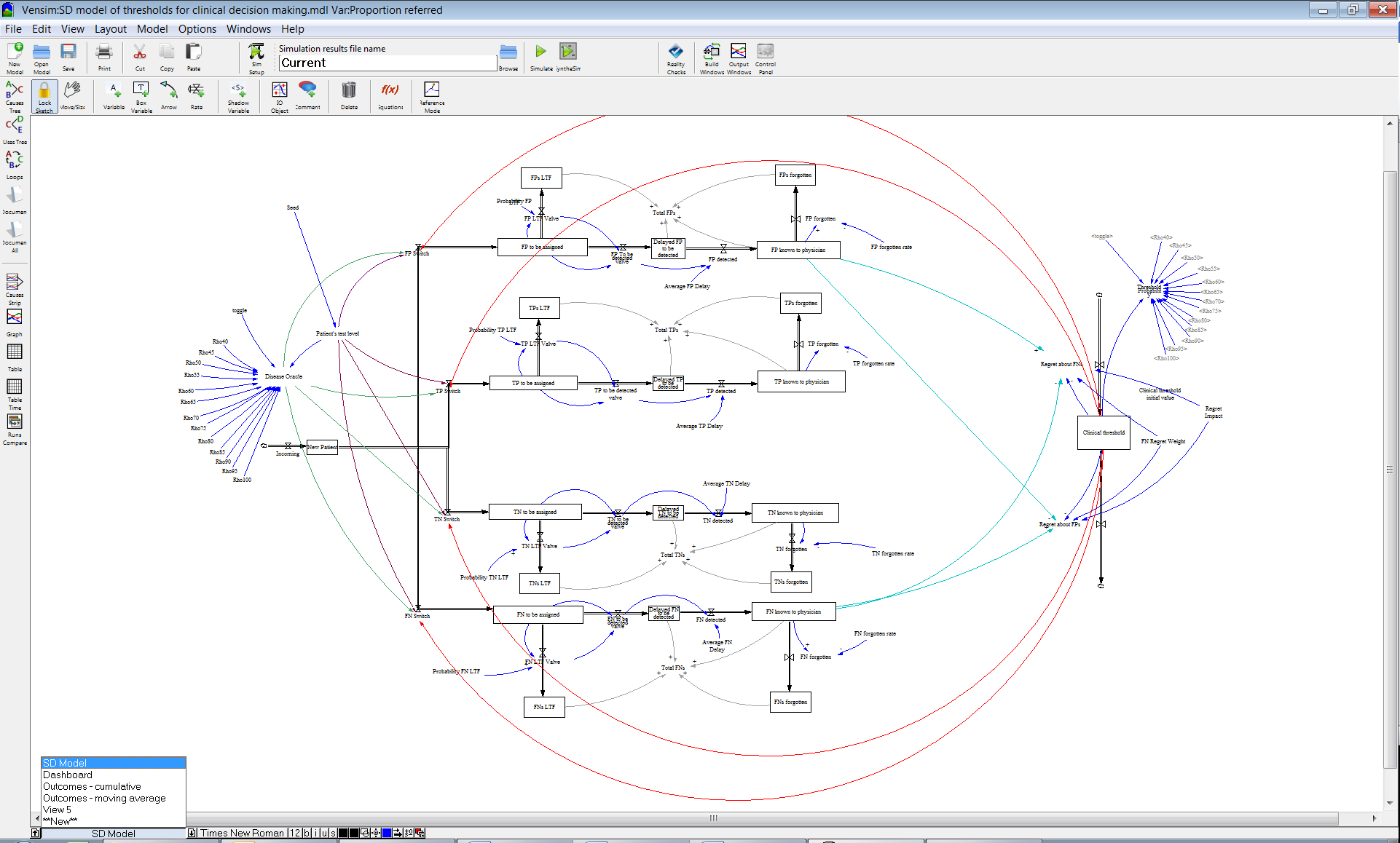


“Equation” button

change to “SD Model” view

- For example, clicking “equation” and then the variable “FP LTF Valve” shows the box below, which includes the equation used to determine whether a false positive result is lost to follow-up


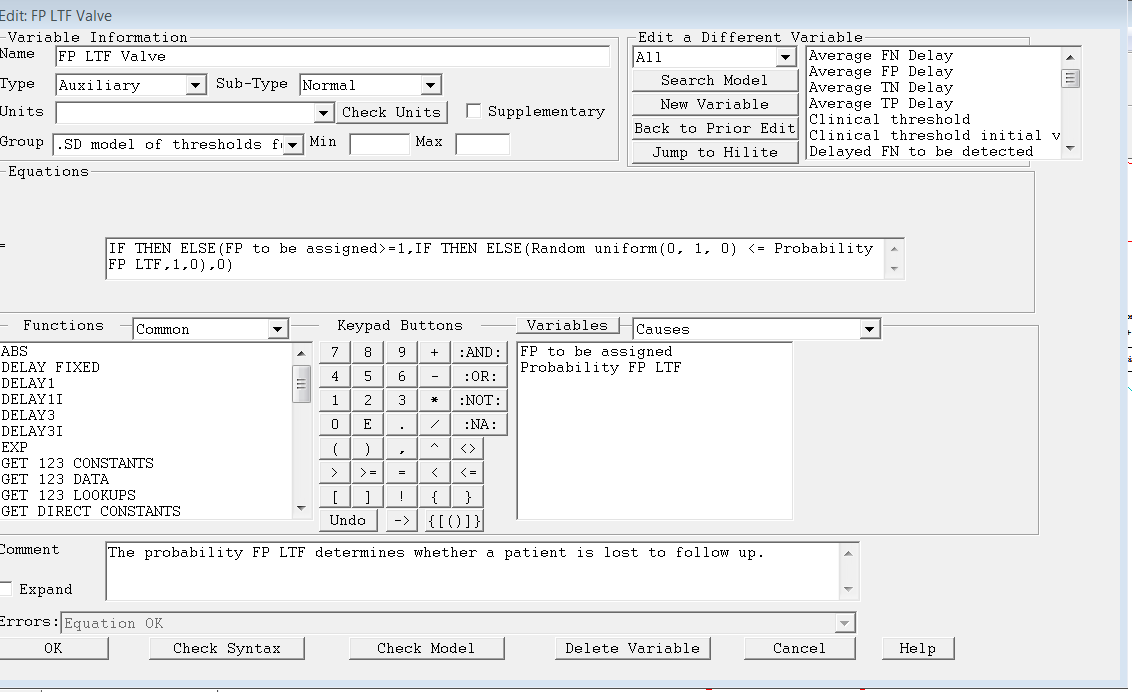


Variables included in equation

Equation

**Brief overview of model structure**

1. New patients enter one at a time
2. Patients are classified as false positive (FP), true positive (TP), true negative (TN), or false negative (FN) depending on:
   1. Patient’s test level (i.e., physician’s assessment of patient symptoms). If *patient’s test level* exceeds *clinical threshold*, then patient is classified as positive
   2. Disease oracle, which calculates the probability of disorder based on a patient’s test level with a random number to determine disease status. Note that probability of disorder is dependent on the accuracy of the assessment, which can vary from rho = .40 to rho = 1.0. In the model, probability is determined via lookup tables which are generated using a separate spreadsheet.
3. Some patients are lost to follow-up (LTF), while others…
4. become known to the physician after a delay, and are subsequently forgotten over time
5. Clinical threshold moves up and down depending on the relative level of regret about false positives (FP) vs. false negatives (FN). Regret is a function of the number of FP or FN results and *FN regret weight*


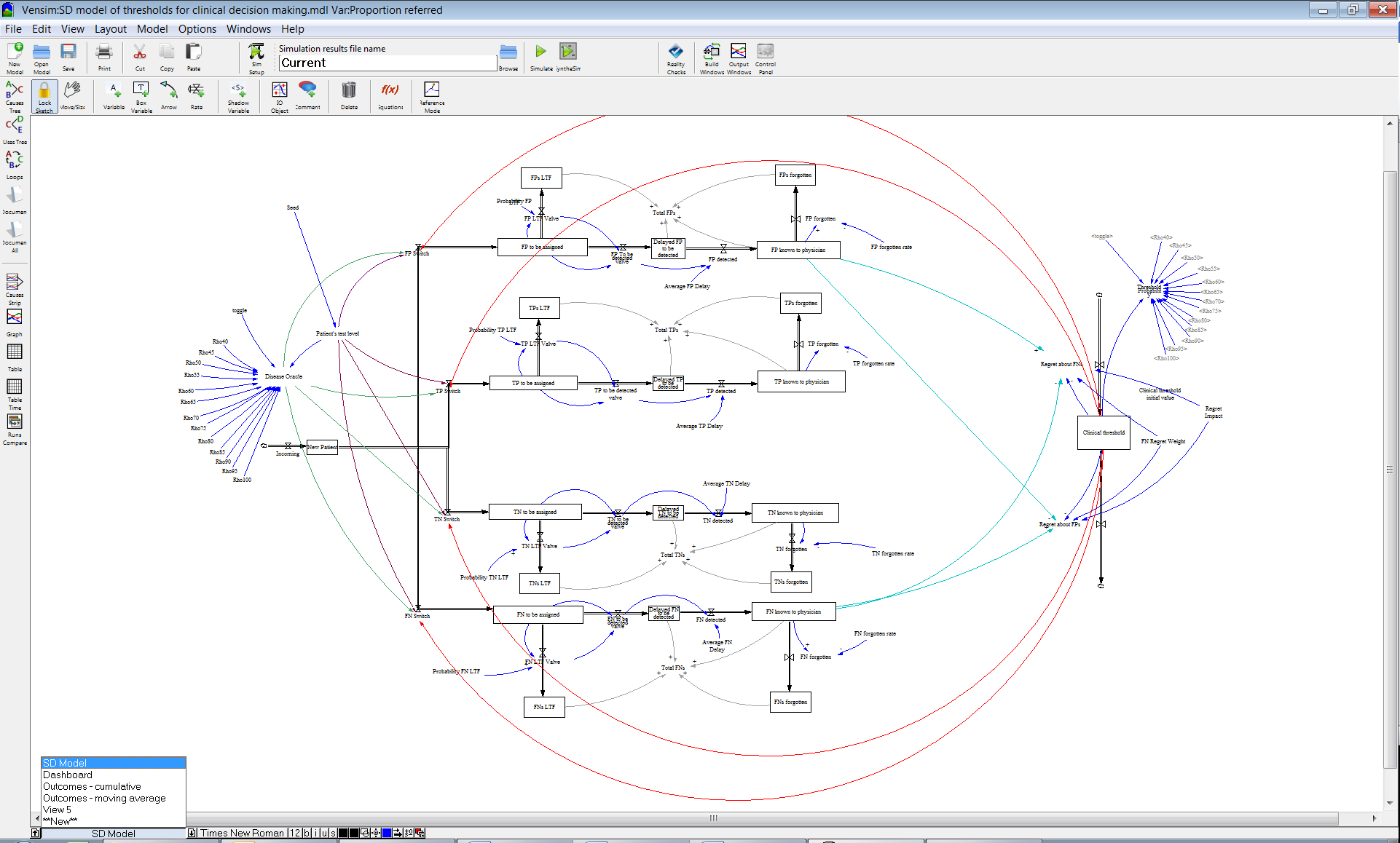


5

1b

1a

4

3

2

1

**Bivariate normal distribution**

The initial assessment and classification of each patient is determined via a randomly generated numbers and a bivariate normal distribution. The bivariate normal distribution is instantiated in excel, which is then used to develop look-up tables for the SD model in Vensim.

- Prevalence is set on the “prevalence of true cases” tab (see below). Symptoms are assumed to be normally distributed in the population. If prevalence is set at 15%, then individuals in the 85^th^ %tile with respect to symptoms will be classified as having disorders.


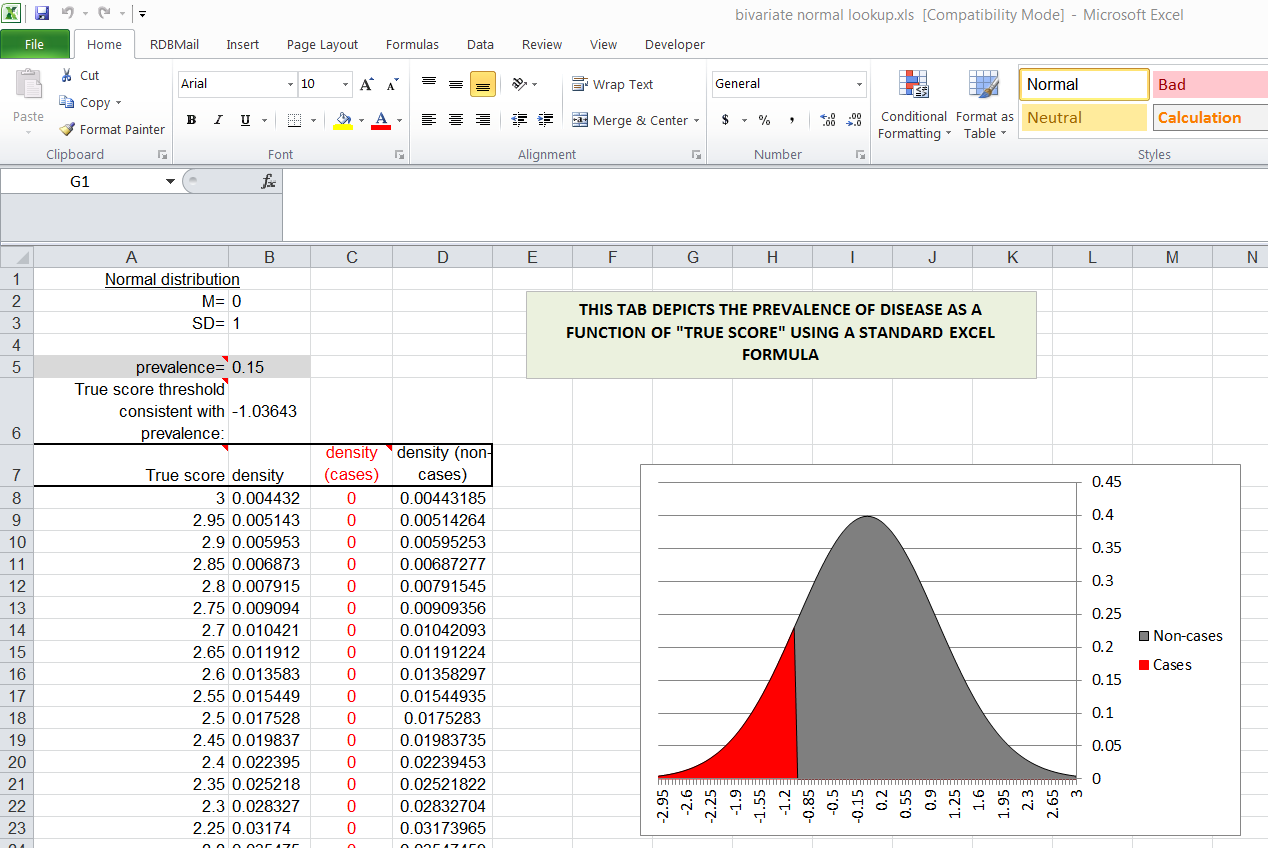


- Physicians do not assess symptoms with perfect accuracy. Therefore, physicians’ perceptions of symptoms are assumed to be correlated with true symptoms with rho ≤ 1. In cell C6 in the figure below (“Lookup” tab), rho is assumed to be .65. Rho reflects the accuracy of the physicians’ assessments.
- For descriptive purposes, the excel chart produces an ROC curve that describes the sensitivity and specificity of every assessment value with respect to disorders as defined above
- In the SD model, physicians’ assessments of patient severity are determined via random draws from a normal distribution. Assuming constant prevalence, for any given assessment of severity, there is a specific probability of truly having a disorder. The tab labeled “Lookup” produces a table of probabilities for a large range of assessment scores (see lookup table in lower left). It also formats the table for Vensim so it can be inserted in the lookup table function of the “rho” variables.


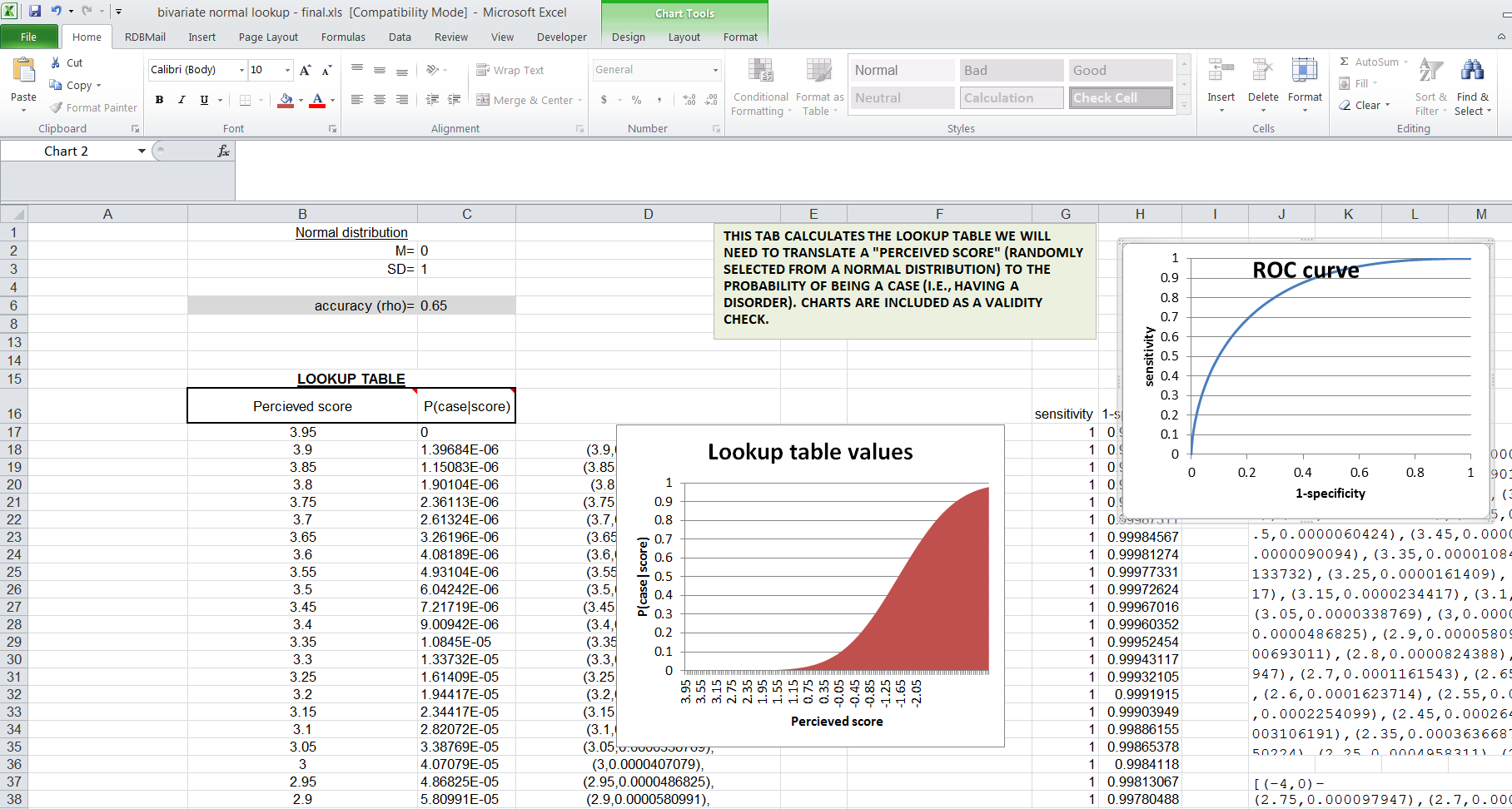


rho

ROC curve

Formatted Lookup table

1. Ventana Systems, Inc.. (2006). *Vensim PLE Software*, Ventana Systems Inc.. Retrieved from Open Educational Resources (OER) Portal at <http://www.temoa.info/node/3682>. Accessed May 30, 2008. [↑](#footnote-ref-1)
