# Supplementary material for: A system dynamics model of clinical decision thresholds for the detection of developmental-behavioral disorders
Source: Implement Sci. 2016 Nov 25;11:156. doi: 10.1186/s13012-016-0517-0 (PMC5123221; doi:10.1186/s13012-016-0517-0)
Supplement: Additional file 4: Table S1. — Supplementary information regarding developmental and behavioral screening trials. (DOC 36 kb) [file 13012_2016_517_MOESM4_ESM.doc]

Appendix table. Supplementary information regarding developmental and behavioral screening trials

|  | | |
| --- | --- | --- |
| **Reference** | **Other interventions/ implementation strategies** | **Other relevant outcomes** |
| Earls et al., 2009 | Screening one element of a "coordinated system…for delivering child development services." Referrals handled by an "early interventionist [who] worked with physicians and parents to…help in dealing with obstacles to intervention and follow-up." | Physician surveys revealed referrals were more likely if multiple domains scored in the at-risk range |
| Schonwald et al., 2009 | Education regarding screening, developmental disabilities; also introduced resources for secondary screening | Referrals to resource for secondary screening appeared to replace referrals to developmental specialists |
| King, 2010 | Screening quality improvement that included "1-day training regarding new terminology, available screening instruments, approaches to practice change, data-collection tools, communication with payers, and collaboration with community-based programs" | Screening rates rose over time; referral rates declined over time; intersite variability documented |
| Guevera et al., 2013 | Three arm study: screening + office support vs screening vs no screening. Office support helped parents complete screeners; assistance with referral unclear | Office support was not associated a change in identification, but it was associated with an increase in referrals |
| Dawson & Camp, 2014 | Screening administered by "trained technician"; no other interventions reported | 70.8% follow-through on referrals |
| Thomas et al., 2016 | None reported | -- |
| Murphy et al., 1996 | No info on colocated care or other interventions--unclear whether any were offered; RA read forms to patients | -- |
| Gall et al., 2000 | "In the state where the study was conducted, a successful partnership between the school-based clinic and managed care plans was formed, and adolescents identified and referred for mental health services received free care" | -- |
| Hacker et al., 2006 | On-site social worker to make referrals; ancillary staff to minimize burden of screening on physicians | -- |
| Stevens et al., 2008 | Computer-support for screening | -- |
| Wintersteen, 2010 | Provider training on youth suicide, including epidemiology, risk and protective factors, assessment, management; on-site social worker to make referrals was not clearly part of intervention | -- |
| Berger-Jenkins et al., 2012 | None reported | Follow-through on referrals increased over time (40%-->83.3%) |
| Rausch et al., 2012 | Providers and support staff recieved “brief introduction” to screening instrument, adolescent depression | Before and after screening, pediatricians surveys revealed "lack of time and paucity of mental health providers" as barriers |
| Jonovich & Alpert-Gillis, 2013 | Screening implemented "as part of quality improvement program"; on-site social worker to make referrals | Follow-through on referrals increased: RR=11.2 (4.4 - 28.6) |
| Romano-Clarke et al., 2014 | Extensive, state-wide provider training and awareness-raising; additional services mental health services implemented concurrently, including Massachusetts Child Psychiatry Access Project, which psychiatric support to primary care physicians | Change in billing for behavioral health services (RR-1.11) suggested that follow-through on referrals increased |
