# Supplementary material for: A system dynamics model of clinical decision thresholds for the detection of developmental-behavioral disorders
Source: Implement Sci. 2016 Nov 25;11:156. doi: 10.1186/s13012-016-0517-0 (PMC5123221; doi:10.1186/s13012-016-0517-0)
Supplement: Additional file 5: Figure S1. — Sensitivity analyses for virtual experiments. (PPTX 385 kb) [file 13012_2016_517_MOESM5_ESM.pptx]

## Slide 1
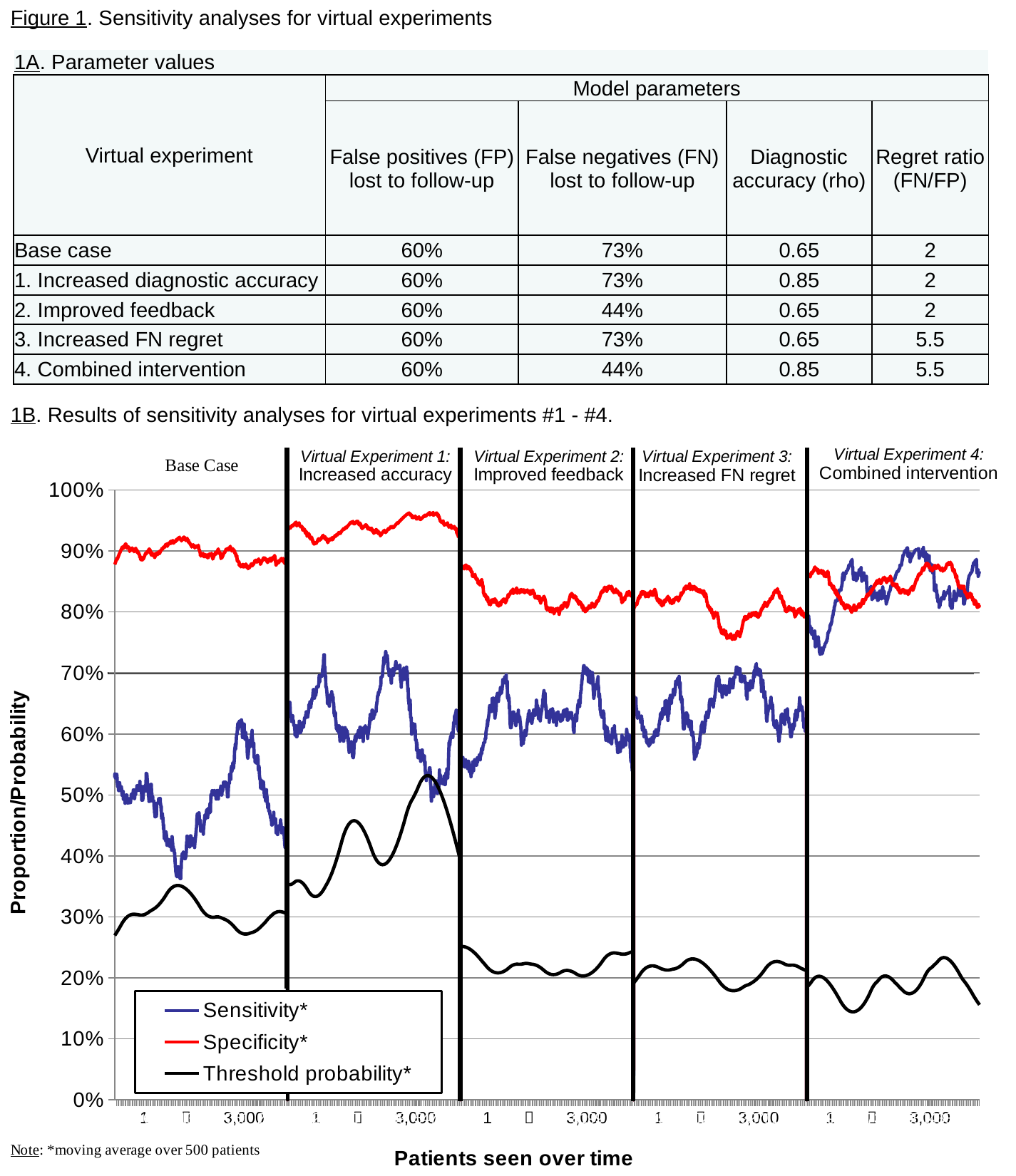

Figure 1. Sensitivity analyses for virtual experiments
| 1A. Parameter values | | | | |
| --- | --- | --- | --- | --- |
| Virtual experiment | Model parameters | | | |
| | False positives (FP) lost to follow-up | False negatives (FN) lost to follow-up | Diagnostic accuracy (rho) | Regret ratio (FN/FP) |
| Base case | 60% | 73% | 0.65 | 2 |
| 1. Increased diagnostic accuracy | 60% | 73% | 0.85 | 2 |
| 2. Improved feedback | 60% | 44% | 0.65 | 2 |
| 3. Increased FN regret | 60% | 73% | 0.65 | 5.5 |
| 4. Combined intervention | 60% | 44% | 0.85 | 5.5 |
1B. Results of sensitivity analyses for virtual experiments #1 - #4.
### Chart
| Category | | | |
|---|---|---|---|1  3,000
1  3,000
1  3,000
1  3,000
1  3,000
